# On the optimal *z*-score threshold for SISCOM analysis to localize the ictal onset zone

**DOI:** 10.1186/s13550-018-0381-9

**Published:** 2018-04-17

**Authors:** Liesbeth De Coster, Koen Van Laere, Evy Cleeren, Kristof Baete, Patrick Dupont, Wim Van Paesschen, Karolien E. Goffin

**Affiliations:** 1 Nuclear Medicine, UZ Leuven, Herestraat 49, 3000 Leuven, Belgium; 2 Nuclear Medicine and Molecular Imaging, Department of Imaging and Pathology, KU Leuven, Herestraat 49, 3000 Leuven, Belgium; 3 Neurology, UZ Leuven, Herestraat 49, 3000 Leuven, Belgium; 4 Department of Neurosciences, KU Leuven, Herestraat 49, 3000 Leuven, Belgium

**Keywords:** Epilepsy, SISCOM, *Z*-score threshold, Ictal SPECT

## Abstract

**Background:**

In epilepsy patients, SISCOM or subtraction ictal single photon emission computed tomography co-registered to magnetic resonance imaging has become a routinely used, non-invasive technique to localize the ictal onset zone (IOZ). Thresholding of clusters with a predefined number of standard deviations from normality (*z*-score) is generally accepted to localize the IOZ. In this study, we aimed to assess the robustness of this parameter in a group of patients with well-characterized drug-resistant epilepsy in whom the exact location of the IOZ was known after successful epilepsy surgery. Eighty patients underwent preoperative SISCOM and were seizure free in a postoperative period of minimum 1 year. SISCOMs with *z*-threshold 2 and 1.5 were analyzed by two experienced readers separately, blinded from the clinical ground truth data. Their reported location of the IOZ was compared with the operative resection zone. Furthermore, confidence scores of the SISCOM IOZ were compared for the two thresholds.

**Results:**

Visual reporting with a *z*-score threshold of 1.5 and 2 showed no statistically significant difference in localizing correspondence with the ground truth (70 vs. 72% respectively, *p* = 0.17). Interrater agreement was moderate (*κ* = 0.65) at the threshold of 1.5, but high (*κ* = 0.84) at a threshold of 2, where also reviewers were significantly more confident (*p* < 0.01).

**Conclusions:**

SISCOM is a clinically useful, routinely used modality in the preoperative work-up in many epilepsy surgery centers. We found no significant differences in localizing value of the IOZ using a threshold of 1.5 or 2, but interrater agreement and reader confidence were higher using a *z*-score threshold of 2.

## Background

A selected group of patients with medically refractory epilepsy, making up to one third of all epilepsy patients, can benefit from epilepsy surgery with a chance of seizure remission in about 60% after temporal lobe resection and about 50% after extratemporal lobe resection [1]. In this subgroup, it is of highest importance to exactly locate the ictal onset zone (IOZ). In addition to other anatomic and functional (imaging) modalities such as video-electroencephalography (EEG) monitoring, magnetic resonance imaging (MRI), ^18^F-FDG positron emission tomography (PET), and others, subtraction ictal single photon emission computed tomography (SPECT) co-registered to MRI (SISCOM) has become a routinely used non-invasive technique in this patient population and it is one of the very few techniques to perform ictal measurements [2, 3].

Earlier studies demonstrated that ictal SPECT plays an important role in providing information during the (peri-) ictal state to locate the IOZ. It has been reported that the correct detection of the IOZ improves by the use of SISCOM versus the conventional side-by-side comparison of ictal and interictal SPECT images (83 vs. 31%) [4]. The accuracy of SISCOM in the localization has been assessed by several studies, comparing SISCOM with invasive ictal EEG, surgical site, or combined modalities [4–6]. Moreover, SISCOM elevates the predictive value of the surgical work-up procedure to preoperatively estimate chances of good surgical outcome [7–10]. Therefore, SISCOM is a highly valuable non-invasive method in the preoperative work-up of these patients if it can be acquired and analyzed properly.

The SISCOM technique consists of a digital subtraction of ictal and interictal perfusion SPECT scans, either by direct subtraction or preferably using more advanced statistical methods with a histogram-based cluster analysis that needs to be thresholded with a certain *z*-score (the number of relative standard deviations compared to no difference). In this statistical analysis, a threshold *z*-value of 2 is generally accepted in most publications [5, 11–14], but this threshold was arbitrarily selected mainly because of lack of hard endpoints (ground truth) and small sample sizes.

The area of highest ictal hyperperfusion is often considered to represent the IOZ. However, apart from statistical noise, also seizure propagation can cause multiple areas of relative hyperperfusion on SISCOM [8]. A higher *z*-score thus results in lower sensitivity and higher specificity compared with a lower threshold [15, 16]. It is thus of greatest importance to properly choose the correct *z*-score in the clinical analysis of SISCOM. Newey et al. studied the optimization of the *z*-score threshold in 26 refractory epilepsy patients who underwent epilepsy surgery with a 6-month seizure-free clinical follow-up and found a slightly improved accuracy with a *z*-score threshold of 1.5 compared to evaluation at the *z*-score threshold of 1 and 2 in the majority of patients [16]. This finding has however not been confirmed.

In this retrospective study, we therefore wanted to investigate whether a *z*-score threshold of 1.5 indeed is more accurate to correctly localize the IOZ than a *z*-score threshold of 2 in a large group of patients with a well-defined drug-resistant epilepsy in whom the location of the IOZ is known since they successfully underwent epilepsy surgery and have been seizure free for at least 1 year.

## Methods

This retrospective study was approved by the local Ethics Committee. All routine investigations were performed according to standard operating procedures in a clinical university hospital setting.

### Patients

Patients were selected from an existing local database of epilepsy patients who underwent either temporal or extratemporal lobe resection from 1998 to 2014. Patients were included in this study if they (1) had undergone both ictal and interictal SPECT-imaging before surgery, (2) had a volumetric preoperative MRI, and (3) were completely seizure free in the postoperative period for at least 1 year after surgery or had less than three seizures in the postoperative period (less than 6 months after surgery) after which they were seizure free for at least 1 year [17]. In all patients, resective surgery strategy and extent of resection were determined by consensus at a patient management meeting with integration of all available clinical information and all paraclinical and technical investigations including neuropsychological evaluation, EEG, structural MRI, language or motor fMRI, FDG PET, and SISCOM. During these meetings, the routinely produced SISCOM with a *z*-score threshold of 2 had been used for clinical interpretation and decision making.

### SPECT injection and imaging

Patients were hospitalized and monitored by video and EEG to direct the ictal injection. ^99m^Tc-ECD (ethylcysteinate dimer) was prepared by kit labeling and injected by epilepsy-trained nurses immediately after clinical seizure onset [18, 19]. Timing of injection was determined on retrospective video and EEG review. Seizure onset was defined as the earliest ictal EEG or clinical evidence of seizure activity. The median injection time was 20.5 s (range 2–116 s), with 73% of patients having injections within 30 s. The median seizure duration was 79 s (range 16–389 s). Three injections in two patients were given post-ictally (two injections in one patient with extratemporal lobe epilepsy, one injection in one patient with temporal lobe epilepsy). In the group of patients with extratemporal lobe epilepsy, the median injection time was 19.5 s (range 1–64 s) with a median seizure duration of 47.5 s (range 16–219 s).

For the interictal study, the patient underwent 5 min of seizure-free EEG monitoring before injection and this monitoring was continued for at least 3 min afterwards. The interictal scan was started 20 min after tracer injection, while ictal scans were acquired between 30 min and 4 h after injection.

SPECT images were acquired using a triple-head Triad XLT (Trionix, Misouri, USA; from 1998 until 2007; protocol one: 120 steps × variable number of seconds (depending on the time between dose calibration and acquisition, 128 × 64 matrix) or a triple-head IRIX Prism (Philips Medical Systems, Cleveland, USA; from 2007 until 2014; protocol two: 120 steps × 15 s, 128 × 128 matrix). Reconstruction of the images was performed using filtered back projection (FBP) with a Butterworth filter at cutoff value of 0.87 cycles/cm and order 7 (protocol one) or using ordered-subset expectation maximization (OSEM) iterative reconstruction with six iterations, eight subsets (protocol two) without postsmoothing. The acquisition and reconstruction was always the same for the ictal and interictal SPECT scans of the same patient.

### SISCOM

Ictal and interictal SPECT studies were co-registered using an automatic registration algorithm based on mutual information using the registration module of Statistical Parametric Mapping (SPM version 8; Wellcome Trust Centre for Neuroimaging, London, UK), implemented in Matlab (R2012; The MathWorks Inc., MA, USA) [20, 21]. The ictal and spatially co-registered interictal images were then normalized for global brain counts within each scan. The transformed, normalized interictal images were subtracted from the normalized ictal image to create an image where the value for each pixel represents the intensity difference between the two data sets. The difference image was smoothed using a 3D-Gaussian smoothing kernel (full width at half maximum = 15 mm) and transformed into a *z*-score using the mean and standard deviation (SD) of the differences in all brain voxels [8, 11, 20]. The average of both SPECT images was co-registered to the structural MRI, and this transformation was applied to the *z*-score image. The thresholded *z*-score map was then co-visualized onto the co-registered preoperative MRI using MRICRO software (Georgia Institute of Technology, Atlanta, GA, USA) for detailed anatomical localization.

### Image readout

Image readout was performed by two experienced nuclear medicine physicians with respectively 10 and 20 years of experience in SISCOM evaluation. Readers were blinded to all clinical information. To score, the brain cortex was divided into 10 volumes of interest (VOI; left and right frontal, temporal, perirolandic, and parietal and occipital cortex), as described previously [6, 16]. The readers were asked to assign, if possible, the IOZ from each individual SISCOM study to one or more of the 10 cerebral VOIs. IOZ was defined as the cortical area with the greatest subtraction values. When the cluster with the highest *z*-score value was located in the occipital lobes, this cluster was disregarded if other significant clusters in the brain were present, since these clusters in the occipital lobe are far more likely to represent a different activation of the visual cortex during the ictal (eyes open) compared to interictal (eyes closed) injection than an IOZ. The two sets of data with *z*-score thresholds of 1.5 and 2 were presented in a randomized order (the reader was only aware of which *z*-score threshold data belonged to). We only investigated thresholds of 1.5 and 2.0 since in the study by Newey et al. and in our clinical experience, *z*-score thresholds of 1.0 give rise to too many false positive clusters.

Readers were also asked to give a confidence level to their localizing score (one = not confident at all to five = very confident). The final localization was based on agreement between the two readers by a consensus read, in case of discrepancy after the first read. In all cases, a consensus agreement between the two readers was found. The study was considered non-localizing if no cluster was found that could fit the IOZ.

The accuracy of SISCOM localization was then evaluated by comparison with the location of surgical resection. If the IOZ determined by the final decision of the SISCOM read encompassed the location of surgical resection, the localization decision was considered correct. In all other cases, it was considered as incorrect, also in the case of a non-localizing SISCOM. The confidence levels between series of SISCOMs (*z*-score 1.5 vs. 2) were analyzed as continuous variables in statistical analysis.

### Statistics

All statistical analyses were performed using SPSS v24 (IBM Business Analytics, Chicago, IL, USA). Significance was determined at the *p* < 0.05 level. With the surgical ground truth as reference, detection ratio was calculated for both thresholds and confidence levels were compared using Wilcoxon signed ranks test. Furthermore, the kappa (*κ*) coefficient and its confidence (SD) were calculated to analyze interrater variability in SPSS.

## Results

### Patient characteristics

Eighty patients (45 male (56%), 35 female (44%)) met the inclusion criteria. Of those, 12 patients underwent two ictal SPECTS, so a total of 92 ictal SPECTs were available for analysis. Demographic and clinical characteristics are shown in Table 1. The mean age at surgery was 38.5 years (SD 11.4 years; range 14–59 years). The preoperative mean seizure burden was 21 seizures/month with 40 patients (50%) having a history of focal to bilateral tonic-clonic seizures. Sixty-one patients had temporal lobe epilepsy (76%), and 19 had extratemporal lobe epilepsy. In patients with extratemporal lobe epilepsy, IOZ was localized in the frontal lobe in 11 patients, in the parietal lobe in 5 patients, in the occipital lobe in 2 patients, and in the insula in 1 patient. Fifty-six (70%) patients had a temporal lobe resection, five patients (6.3%) had a temporal lesionectomy, one patient (1.3%) had temporal radiosurgery, and 11 patients (13.8%) had a lesionectomy in the frontal lobe, two patients (2.5%) in the occipital lobe, and five patients (6.3%) in the parietal lobe. Histopathological analysis was available in 78 patients and showed hippocampal sclerosis in 46 patients, focal cortical dysplasia in 25 patients, dysembryogenic neuro-endocrine tumor (DNET) in 4 patients, cavernous hemangioma or no abnormality in 3 patients, dual pathology or reactive astrogliosis in 2 patients, and ganglioglioma, epidermoid cyst, meningioma, oligodendroglioma, neurocytoma, or unclear findings in 1 patient. After surgery, all patients were seizure free for at least 1 year. In seven patients, one to three seizures were present in the immediate postoperative period but they were seizure free afterwards for the rest of their follow-up, which was at least 1 year.

**Table 1:** Patient demographics and clinical characteristics (mean ± SD)

	Mean (± SD)
Age (years)	38.5 (11.4)
Male/female	45 M/35 F
Seizure burden (number of seizures per month)	21.5 (43.9)
Ictal SPECT injection time (seconds)	27.1 (23.6)
Seizure duration (seconds)	91.7 (62.9)
TLE/nTLE	61/19

*TLE* temporal lobe epilepsy, *nTLE* extratemporal lobe epilepsy

### Comparison of IOZ localization between *z*-score threshold of 1.5 and 2

At *z*-score threshold of 1.5, 1/92 scans was considered non-localizing vs. 4/92 scans at *z*-score threshold of 2.

**Fig. 1:**
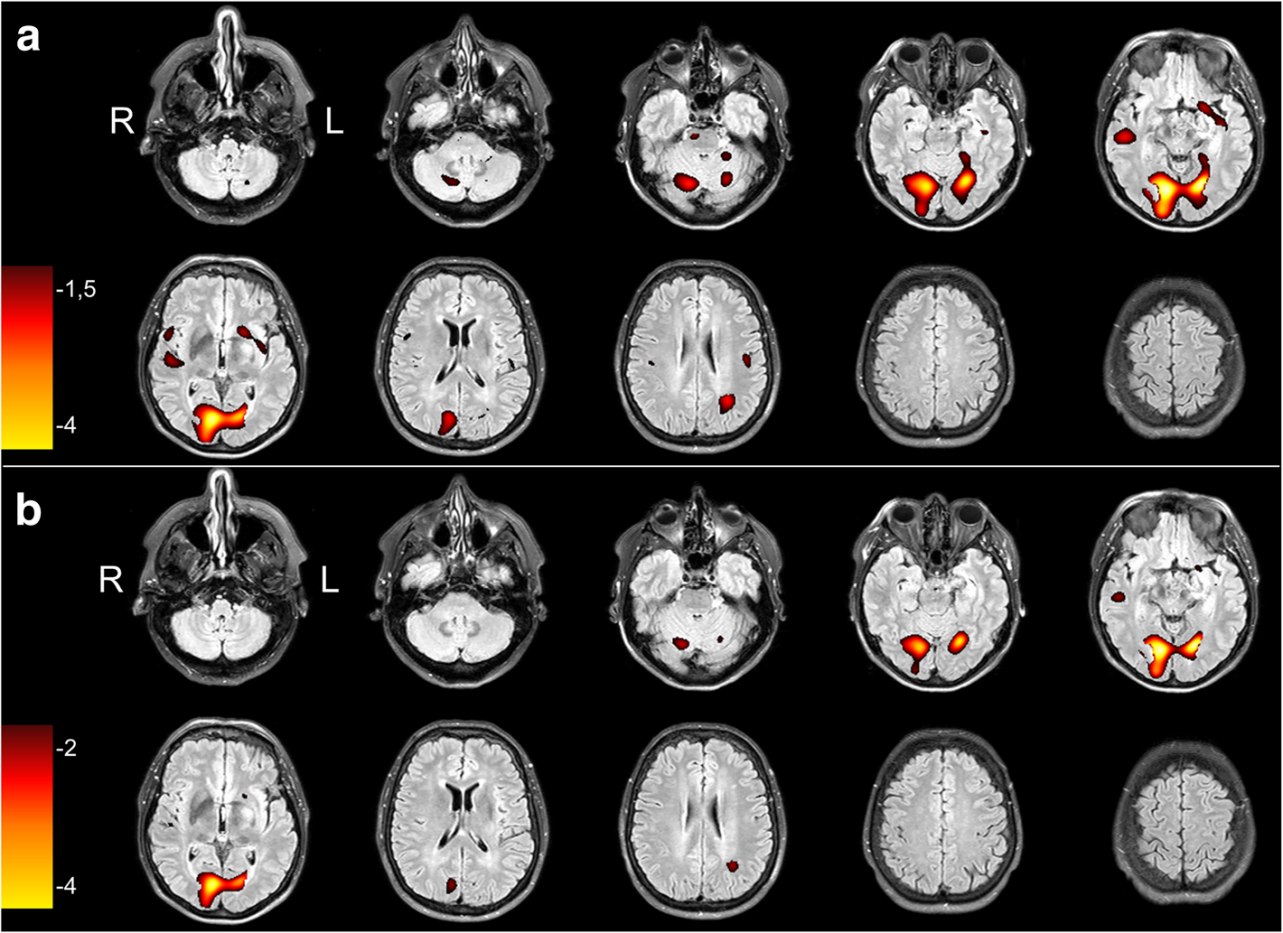
Representative transverse SISCOM images of a 53-year-old patient with clinically temporal lobe epilepsy caused by an epidermoid tumor in the left occipital lobe, at the base of the skull at *z*-score thresholds of 1.5 and 2, superimposed on the preoperative MRI. Images are in radiological orientation. **a** The images at threshold of *z* = 1.5 show a widespread hyperperfusion in both occipital cortices but also a significant cluster in the right temporal lobe which was interpreted as the ictal onset zone with propagation to both occipital cortices. **b** The images at threshold of *z* = 2 show a clear focal hyperperfusion in the left occipital lobe with propagation towards the contralateral occipital lobe. The cluster in the right temporal lobe was much smaller and was interpreted as insignificant and caused by noise

At a *z*-score threshold of 1.5, 70% (64/92) of the studies were correctly localized by the consensus read. This value increased to 72% (66/92) at a *z*-score threshold of 2 (*p* = 0.17). An example of a typical patient is shown in Fig. 1. The rate of correct localization was significantly higher in patients with temporal versus extratemporal epilepsy at a *z*-score threshold of 1.5 (81 vs. 40%, *p* < 0.001) as well as at a *z*-score threshold of 2 (81 vs. 48%, *p* = 0.004). Within each group, however, there was no significant difference in rate of correct localization between a *z*-score threshold of 2 and 1.5.

Consensus read was necessary in 23 of the 92 SISCOMs analyzed with a *z*-score threshold of 1.5 and in 18 of the 92 SISCOMs analyzed with a *z*-score threshold of 2. Analysis of interrater reliability analysis demonstrated no significant differences in the correctness of the IOZ localization between a *z*-score threshold of 1.5 versus a *z*-score threshold of 2 for any reviewer (reader one: 63/92 at threshold 1.5 vs. 64/92 at threshold 2; reader two: 64/92 vs. 66/92). However, the reviewer with 20 years of experience had a significantly higher confidence level using a *z*-score of 2 vs. 1.5 (4.2 ± 1.1 vs. 4.0 ± 1.0, *p* = 0.002), in contrast to the reviewer with 10 years of experience (4.3 ± 1.0 vs. 4.2 ± 1.1, *p* = 0.39). The agreement in localization between the two reviewers was moderate at a *z*-score of 1.5 (*κ* = 0.69 ± 0.08—concordance in 81 out of 92 reads) and significantly better at a *z*-score of 2 (*κ* = 0.84 ± 0.06—concordance in 86 out of 92 reads; *p* < 0.001).

**Fig. 2:**
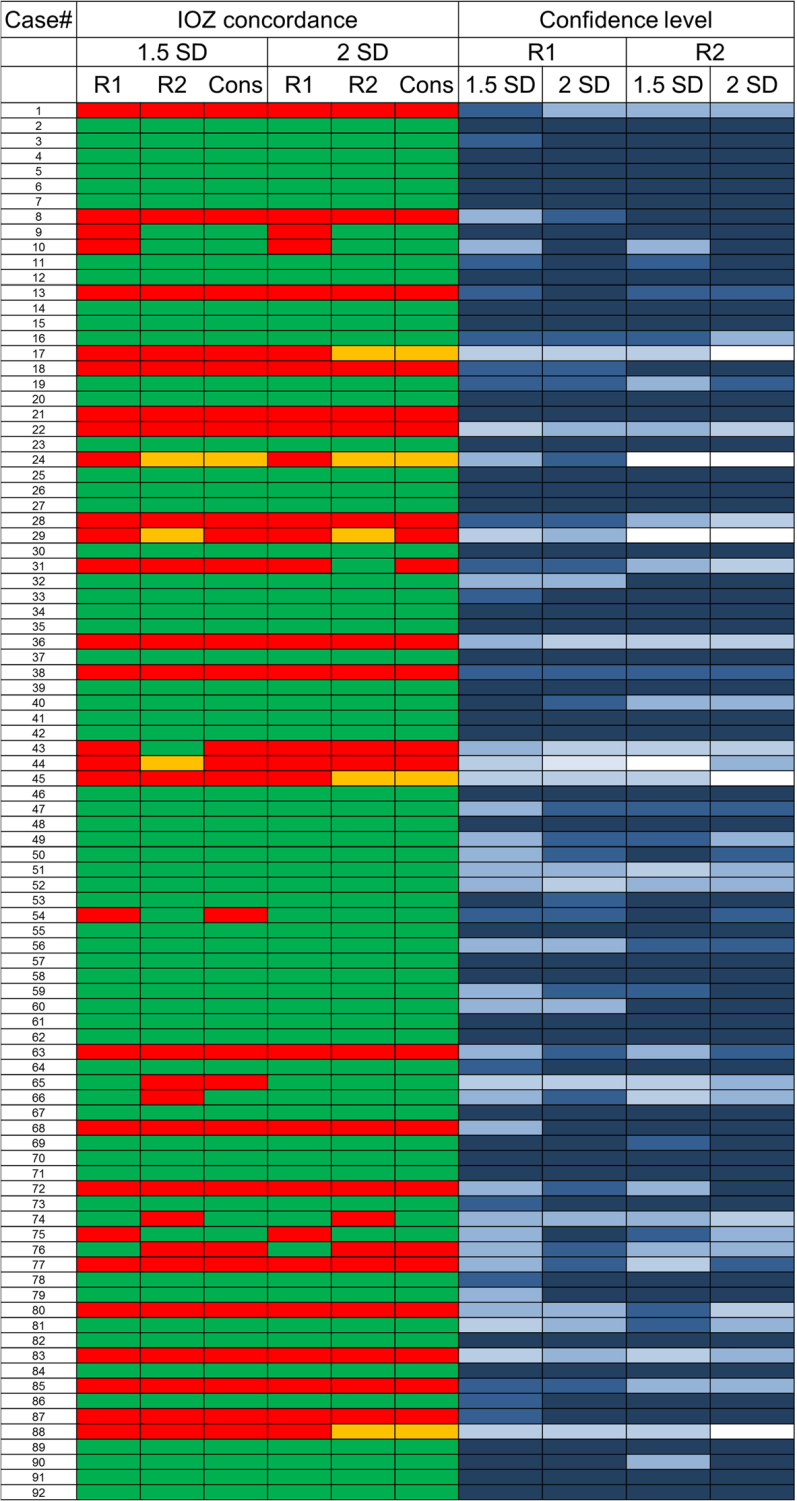
Reader consensus table for the 92 analyzed SISCOM images. Green = concordance with surgery localization, red = different region, and orange = non-localizing at threshold of *z* = 1.5 SD and *z* = 2 SD. Reader confidence level (light blue = 1 to darkest blue = 5). Reader one (R1) = 20 years of experience, reader two (R2) = 10 years of experience, Cons: consensus read

Figure 2 shows a color-coded overview for both readers, the consensus read at *z*-score thresholds of 1.5 and 2, and reader confidence values.

Again, the confidence level was significantly higher in temporal lobe epilepsy compared to extratemporal lobe epilepsy for both reviewers at a *z*-score threshold of 1.5 (reviewer 1 4.2 vs. 3.4, *p* = 0.002; reviewer 2 4.4 vs. 3.6, *p* = 0.001) as well as at a *z*-score threshold of 2 (reviewer 1 4.4 vs. 3.6, *p* = 0.001; reviewer 2 4.6 vs. 3.7, *p* < 0.001). Within these subgroups, there was a significantly higher confidence level in temporal lobe epilepsy for reviewer 1 at a *z*-score threshold of 2 vs. 1.5 (4.4 vs. 4.2, *p* = 0.02). Confidence levels were also higher in extratemporal lobe epilepsy for both reviewers and in temporal lobe epilepsy for reviewer 2, but these differences failed to reach significance.

## Discussion

SISCOM is a valuable, non-invasive tool in determining the IOZ in presurgical work-up for medically refractory epilepsy patients who are candidates for curative surgery. In clinical practice, a *z*-score threshold of 2 is generally accepted to define the cluster implicating the IOZ [5, 11–14]. This cutoff value of 2 SD was arbitrarily selected, and its robustness has not been clearly assessed. Newey et al. evaluated the localizing value of four different *z*-score thresholds (1, 1.5, 2, and 2.5) in a group of 26 patients, who were at least 6 months seizure free after surgery and showed that SISCOM images were more accurately analyzed using different *z*-scores on an individual basis, with in the majority of cases an optimal *z*-score of 1.5 [16]. At a *z*-score threshold of 1.5, 77% of the studies were correctly localized, while at a *z*-score threshold of 2, only 50% of the studies were correctly localized.

Our study, which included a larger group of 80 patients with 92 scans, did not confirm a clear advantage of using a *z* > 1.5 threshold since there was no difference in correct localization between the two evaluated values (72 vs. 70%). These slightly discrepant findings may be due to a different scoring protocol and region definition in their study and to a different method of cluster determination. Furthermore, our study contained a larger portion of patients with temporal lobe epilepsy (74 vs. 50% in Newey et al. [16]). Since it is known that the interpretation of ictal SPECT is more challenging in mostly brief extratemporal seizures with early propagation of ictal activity and an earlier switch from ictal hyperperfusion to postictal hypoperfusion [22], the larger group of patients with temporal epilepsy may explain a better detection rate for both thresholds. There were no major differences in age between both groups (38.5 ± 11.4 years in our study vs. 36.5 ± 15.2 years in Newey et al.). However, there were differences with lower seizure burden in our study (5.0 seizures/week vs. 9.5), history of generalized motor seizures (50 vs. 69%), and a slight male preponderance (56% in our study vs. 35%).

Regarding interrater agreement at the two *z*-score thresholds, Newey et al. reported moderate *κ* coefficients of 0.7 and 0.6 at *z*-score thresholds of 1.5 and 2, respectively. At the 1.5 threshold, this was very similar to our study (kappa = 0.69), but significantly lower than the value found at 2 (*κ* = 0.84). Also here, the higher proportion of easier detectable temporal lobe epilepsy in our study population may be responsible for this difference.

Regarding reader confidence, only one reader felt significantly more confident (*p* = 0.002) analyzing the SISCOM data with a *z*-score of 2 than with a *z*-score of 1.5, due to the smaller number of hyperperfusion clusters and less chance of false positive calls.

Overall, both a *z*-score threshold of 1.5 and 2 have a similar accuracy for localizing the IOZ in SISCOM analysis. However, since the kappa coefficient in our study was significantly (*p* < 0.001) higher at a *z*-score of 2, these results do suggest that the *z*-score threshold of 2 remains the most optimal value for use in clinical routine, irrespective of reader experience. Although not explicitly investigated in this study, it may however be advantageous to use a combination of 1.5 and 2 as threshold, or even scanning over a variable threshold value as can be done in current software, to get a better sense for the location of hyperperfusion clusters related to the IOZ and the spreading pattern.

An inherent limitation of this study is its retrospective character. All patients included were selected out of a large database of epilepsy patients on the basis of the inclusion criteria of this study, so this group might not be a perfect representation of all epilepsy patients that are candidates for epilepsy surgery and undergo ictal SPECT. In previous studies, the localizing value of SPECT was often compared with seizure foci suggested by other localizing information such as EEG and MRI. However, this makes it difficult to evaluate if incorrect SISCOM localization was truly incorrect or if it was due to incorrect localization of the seizure focus by other modalities. To rule out this bias, we only included patients who were seizure free at least 1 year after epilepsy surgery, so that the localizing value of the SISCOM is compared to the surgical resection area. However, this entails that the selection of seizure-free patients might involve a bias by itself. Especially cases with discordant clinical, EEG and imaging data may not be selected for elective surgery and therefore represent either more difficult interpretation or very small or absent SISCOM clusters. Based on the study of Newey et al. [16] as well as based on experience from extensive use of SISCOM in clinical routine, we chose not to evaluate a *z*-score threshold of neither 1 SD, because it shows too much propagated activity, nor 2.5 SD because it is too stringent and only shows clusters of very intense relative hyperperfusion.

## Conclusions

We found no difference in correct ictal onset zone localization by means of SISCOM analysis when using *z*-score thresholds of 1.5 or 2 in an extensive set of well-characterized patients who are seizure free after epilepsy surgery. As interrater agreement and observer confidence are higher at the *z*-score threshold of 2, we advocate that this should be used in the routine clinical setting.

## Abbreviations

DNET: Dysembryogenic neuro-endocrine tumor
ECD: Ethylcysteinate dimer
EEG: Electroencephalography
FBP: Filtered back projection
IOZ: Ictal onset zone
MRI: Magnetic resonance imaging
OSEM: Ordered-subset expectation maximization
PET: Positron emission tomography
SD: Standard deviation
SISCOM: Subtraction ictal SPECT co-registered to MRI
SPECT: Single photon emission computed tomography
